# Draft Genome Sequences of Exfoliative Toxin A-Producing *Staphylococcus aureus* Strains B-7772 and B-7777 (CC8/ST2993) and B-7774 (CC15/ST2126), Isolated in a Maternity Hospital in the Central Federal District of Russia

**DOI:** 10.1128/genomeA.00064-16

**Published:** 2016-03-03

**Authors:** Igor Abaev, Yury Skryabin, Angelina Kislichkina, Alexandr Bogun, Olga Korobova, Nadezhda Mayskaya, Igor Shemyakin, Ivan Dyatlov

**Affiliations:** State Research Center for Applied Microbiology and Biotechnology, Obolensk, Russia

## Abstract

*Staphylococcus aureus* clonal complex 8 (CC8) has not been associated with staphylococcal scalded-skin syndrome (SSSS) in newborns and exfoliative toxin genes. Here, we report the draft genome sequences of exfoliative toxin A-producing B-7772, B-7777 (both CC8), and B-7774 (CC15) strains associated with SSSS in newborns.

## GENOME ANNOUNCEMENT

*Staphylococcus aureus* causes a wide range of syndromes (1). Definite staphylococcal infections are mainly associated with a few *S. aureus* genetic backgrounds. Exfoliative toxin produced by *S. aureus* is the causative agent of staphylococcal scalded-skin syndrome (SSSS) in young children (2). SSSS in newborns is mainly associated with *S. aureus* strains of clonal complex 121 (CC121) and CC15, which carry exfoliative toxin A (*eta*) and/or exfoliative toxin B genes (3, 4). The *eta* gene is carried by ETA-converting bacteriophages of the family *Siphoviridae* (5, 6). CC8 strains were not previously associated with the *eta* gene and SSSS in newborns.

We announce here the draft genome sequences of CC15 and CC8 strains isolated during two outbreaks in a maternity hospital (Belgorod, Russia) in 2013 and 2014. In 2013, CC15 strain B-7774, belonging to sequence type 2126 (ST2126), was recovered from infected newborns, and isolates of CC8/ST2993 strain B-7772 carrying the *eta* gene were obtained from nasal samples from hospital personnel. CC8/ST2993 strain B-7777, which is identical to strain B-7772, was recovered from infected newborns in 2014, suggesting the replacement of the traditional CC15/ST2126 strain. The aim of this work was to obtain whole-genome sequences of isolated strains for further analysis.

*S. aureus* genomic DNA was extracted with cetyltrimethylammonium bromide (CTAB) (7). Whole-genome shotgun sequencing was performed using the 318 Chip and 400-bp chemistry on an Ion Torrent PGM platform. A total of 737,623, 1,171,947, and 525,784 reads with mean read lengths of 252 bp, 272 bp, and 277 bp and coverages of 64×, 128×, and 44× for strains B7772, B7774, and B7777, respectively, were generated. All reads were *de novo* assembled by Newbler 2.9 (Roche). The draft genome assemblies for strains B7772, B7774, and B7777 included 79, 71, and 59 contigs >200 bp, respectively. Annotation was carried out using the NCBI PGAP annotation pipeline (http://www.ncbi.nlm.nih.gov/genome/annotation_prok/). The assemblies for strains B7772, B7774, and B7777 contained 2,748, 2,625, and 2,714 predicted coding sequences, respectively. The total lengths of the contigs for each genome were 2,777,063 bp, 2,705,467 bp, and 2,778,291 bp, respectively, with a G+C content of 32.7%.

Prophage regions were revealed using PHAST (8). Strains B7772 and B7777 carried two intact prophage regions. The first region was 41,167 bp in length, with a G+C content of 34.9%, and it contained the *eta* gene. The second region was 42,522 bp in length, with a G+C content of 33.1%. Strain B7774 harbored one complete *eta*-encoding prophage, with a size of 42,061 bp and G+C content of 35.1%. Plasmids were identified using PlasmidFinder (9). Strains B7772 and B7777 carried a plasmid of about 27 kb, with a G+C content of 27.1%. Strain B7774 contained a plasmid of about 20 kb, with a G+C content of 28.3%.

### Nucleotide sequence accession numbers.

The draft genome sequences of the *S. aureus* strains B7772, B7774, and B7777 have been deposited at DDBJ/EMBL/GenBank under the accession numbers LJBK00000000, LJBL00000000, and LJBM00000000, respectively. The versions described in this paper are the first versions, LJBK01000000, LJBL01000000 and LJBM01000000.
